# Exposure to Inflammatory Cytokines IL-1β and TNFα Induces Compromise and Death of Astrocytes; Implications for Chronic Neuroinflammation

**DOI:** 10.1371/journal.pone.0084269

**Published:** 2013-12-19

**Authors:** Christa van Kralingen, Dan Ting Kho, Jessica Costa, Catherine Elizabeth Angel, E. Scott Graham

**Affiliations:** 1 Centre for Brain Research, School of Medical Sciences, Faculty of Medical and Health Sciences, University of Auckland, Auckland, New Zealand; 2 School of Biological Sciences, Faculty of Sciences, University of Auckland, Auckland, New Zealand; Emory University, United States of America

## Abstract

**Background:**

Astrocytes have critical roles in the human CNS in health and disease. They provide trophic support to neurons and are innate-immune cells with keys roles during states-of-inflammation. In addition, they have integral functions associated with maintaining the integrity of the blood-brain barrier.

**Methods:**

We have used cytometric bead arrays and xCELLigence technology to monitor the to monitor the inflammatory response profiles and astrocyte compromise in real-time under various inflammatory conditions. Responses were compared to a variety of inflammatory cytokines known to be released in the CNS during neuroinflammation. Astrocyte compromise measured by xCELLigence was confirmed using ATP measurements, cleaved caspase 3 expression, assessment of nuclear morphology and cell death.

**Results:**

Inflammatory activation (IL-1β or TNFα) of astrocytes results in the transient production of key inflammatory mediators including IL-6, cell surface adhesion molecules, and various leukocyte chemoattractants. Following this phase, the NT2-astrocytes progressively become compromised, which is indicated by a loss of adhesion, appearance of apoptotic nuclei and reduction in ATP levels, followed by DEATH.

The earliest signs of astrocyte compromise were observed between 24-48h post cytokine treatment. However, significant cell loss was not observed until at least 72h, where there was also an increase in the expression of cleaved-caspase 3. By 96 hours approximately 50% of the astrocytes were dead, with many of the remaining showing signs of compromise too. Numerous other inflammatory factors were tested, however these effects were only observed with IL-1β or TNFα treatment.

**Conclusions:**

Here we reveal direct sensitivity to mediators of the inflammatory milieu. We highlight the power of xCELLigence technology for revealing the early progressive compromise of the astrocytes, which occurs 24-48 hours prior to substantive cell loss. Death induced by IL-1β or TNFα is relevant clinically as these two cytokines are produced by various peripheral tissues and by resident brain cells.

## Introduction

Astrocytes comprise a heterogeneous population of cells within the central nervous system (CNS) [1]. They are integral to maintaining a functional blood-brain barrier (BBB), providing trophic support to neuronal cells, are involved in neurotransmission events, and have a well-recognised role in innate immune surveillance throughout the CNS [2,3]. All brain regions contain astrocytes and, along with microglia and the frequently understated endothelial cells, make up an important complementary trio, which orchestrate local inflammatory responses to acute insults (such as death of resident brain cells or vascular damage). 

In addition, the astrocytes that are part of the BBB are amongst the first cells to encounter blood-derived leukocytes entering the brain during certain types of neuroinflammatory insult [3]. Increased leukocyte diapedesis occurs in neurological conditions such as stroke or multiple sclerosis. As such, astrocytes are strategically located to influence direct interactions with leukocytes or interaction with endothelial cells of the BBB [3].

Astrocytes are an important source of cytokines, both neurotrophic and inflammatory, and have the capacity to respond to a variety of cytokines themselves [4]. In their resting state, astrocytes express a repertoire of receptors for inflammatory cytokines (IL-1β and TNFα), chemokines and danger signals (including TLR ligands)[5], with numerous other receptors and inflammatory mediators being induced following the appropriate activation signals [6] from other resident brain cells, infiltrating leukocytes or invading pathogens [2]. Overall, the CNS is now widely recognised as an immunosuppressed environment, with very low numbers of surveying leukocytes being present in healthy states [2]. These are largely restricted to T lymphocytes, which reside within the cerebrospinal fluid [2]. Leukocytes are actively excluded from the healthy brain by the blood-brain barrier and blood-cerebrospinal fluid barriers, of which astrocytes are an integral part [7]. 

With these roles in mind, it is easy to envisage that astrocyte health is a critically important factor for long-term brain health. Previously we used cytometric bead array technology to screen the secretory output from astrocytes following inflammatory activation [8]. We found that astrocytes produced numerous monocyte and neutrophil chemoattractants at biologically relevant concentrations, as well as potent pro-inflammatory mediators like IL-6 and TNFα, and the anti-inflammatory mediator IL-13 [8]. During the previous study, we noted evidence of astrocyte death following IL-1β and TNFα treatment. However, this was only apparent after at least 48 hours post cytokine addition. It was the aim of this study to investigate the time-line of astrocyte compromise relative to cytokine-mediated stimulation and to prove whether astrocyte death consistently occurred following inflammatory activation. 

## Methods

### Differentiation of NT2 astrocytes

All media, serum and antibiotics were purchased from Invitrogen. The Ntera2/D1 cell line was purchased from ATCC. NT2-astrocytes were produced using a 10-11 week differentiation protocol described in detail previously [9-11]. A critical step required to improve astrocyte purity is the double-harvesting of the differentiated neurons at week 6 to 7 [12]. This ensures a final yield of NT2-astrocytes with few contaminating neuronal cells. NT2-astrocytes were cultured in DF10 media (DMEM/F12 supplemented with 10% FBS and 50U/mL penicillin and 50μg/mL streptomycin). The NT2 astrocytes were cryopreserved following differentiation using 45% FBS, 10% DMSO and 45% DMEM/F12. 

### Culture and Stimulation of NT2-Astrocytes

Astrocyte cultures were grown in T75 maintenance flasks (Nunc) for up to one week prior to use. Cells were trypsinised, counted and seeded into 96 well plates (Nunc plates provide better adhesion). 6500 cells were seeded in 100 µl DF10 and allowed to recover at 37°C for 24 hour prior to drug treatments, as determined previously [11]. Typically, drugs were prepared as 2x and the cells were stimulated for the indicated period. Cytokines used for stimulating the NT2-astrocytes were purchased from PeproTech (New Jersey, US). 

### Intracellular staining in NT2-astrocytes

NT2-astrocytes were seeded and stimulated as described above and fixed with 4% paraformaldehyde. After permeabilisation with PBS containing 0.1% triton X-100, cells were incubated with primary antibodies overnight at 4°C in immunobuffer (PBS containing 1% goat serum). The primary antibodies were for vimentin (Abcam ab15248; rabbit polyclonal 1:1000 dilution); glial fibrillary acidic protein (GFAP from DAKO Z0334, rabbit polyclonal; 1:1,000); Cleaved-caspase 3 (Cell Signalling Technologies #9661; rabbit polyclonal 1:1000 dilution) and IP-10 (Abcam; rabbit polyclonal; 1:1000). Secondary antibodies were purchased from Molecular Probes. For immunofluorescence, species-specific Alexa488 conjugated antibodies were used at 1:400 dilution for 2 hours at ambient. For bright-field imaging, biotinylated secondary antibodies were used at 1:400 for 2 hours at room temperature. The tertiary step was conducted using ExtrAvidin-HRP (Sigma) and DAB to form a brown colour precipitate. This latter method was favoured over fluorescence to provide a better visualisation of the astrocyte processes and cytoplasm. Nuclei were counter-stained using Hoechst 33258. Imaging was conducted using a Leica DMR microscope mounted with a DFC425-C camera and images acquired using Leica LASCore software. 

### Phenotypic analysis of differentiated astrocytes

Harvested astrocytes were routinely stained for MAP 2, GFAP and vimentin (see table 1). The differentiated NT2 astrocytes are vimentin and GFAP positive, but negative for MAP 2, which is expressed specifically by neuronal cells. In our experience, the cryopreservation of the astrocytes kills the few neuronal cells that make it to the final stages of differentiation. Astrocyte cultures were used that were >95% GFAP^+^/vimentin^+^ and lacked MAP 2. 

**Table 1:** Summary of antibodies and CBA flex sets used throughout this study.

**Antibody**	**Cat #**	**Host**	**Usage**
Vimentin	Abcam #15248	Rabbit	1:1000
GFAP	DAKO #Z0334	Rabbit	1:1000
IP-10	Abcam #9807	Rabbit	1:1000
Cleaved caspase -3	Cell Signaling Tech # 9661	Rabbit	1:1000
MAP 2	Sigma #M4403	Mouse IgG_1_	1:1000
Anti-rabbit IgG Alex488	Molecular Probes	Goat	1:400
Anti-rabbit IgG biotinylated	Sigma B7389	Goat	1:400
ExtrAvidin HRP	Sigma E2886	NA	1:250
**CBA flex set**	**BD Cat #**	**Beadposition**	
IL-6	558276	A7	
IL-8	558277	A9	
sCD54/ICAM-1	560269	A4	
sCD106/VCAM	560427	D6	
IP-10	558280	B5	
MCP-1	558287	D8	
RANTES	558324	D4	
MIP1α	558325	B9	

The antibody source, product code, host species and working dilution are detailed. For the CBAs, the relative bead position and product code are detailed.

### Measurement of secreted cytokines using cytometric bead arrays

Cells were seeded into 96 well plates at 6500 astrocytes per well and allowed to recover overnight. The following day cytokines were prepared as 1x (5ng/mL). The media was fully aspirated (gently) from each well and then fresh media with or without the cytokine was added for the designated period-of-time. At the end of the experiment, the conditioned media was gently aspirated and transferred to a new 96 well plate and centrifuged to remove any cellular debris (350 x*g* for 5 minutes). Then single-use aliquots (40 µl) were prepared and stored at -20°C until required. Of importance, aliquots were only ever thawed once. The concentration of specific inflammatory cytokines present in astrocyte-conditioned media was measured using cytometric bead array (CBA; BD Biosciences, USA) as described previously [8]. This assay is multiplexed and measures the concentration of each cytokine simultaneously. CBAs were analysed using an Accuri C6 flow-cytometer fitted with the appropriate FL3 selectable laser module. The FCS data files were exported and analysed using FCAP-array software (version 3.1; BD Biosciences), which converts the raw fluorescent intensity values into concentration using a standard curve for each cytokine measured. Graphs were plotted using GraphPad Prism. Details of the flex sets are listed in table 1. 

### ATP measurements

Cell viability was measured using the ATPlite assay (Perkin Elmer) [13]. For all experiments a new standard curve was generated using cells serially diluted 2-fold from 20,000 to 0 (20, 000, 10,000, 5000, 2500, 1250, 625, 312, 156, 78, 39, 20, 10, 0). The manufacturer’s protocol was followed precisely and the luminescence levels were measured with a VICTOR X Light (Perkin Elmer) plate reader. GraphPad Prism was used to calculate the gradient co-efficient for calculation of cell numbers for all samples. In all experiments, the R^2^ value for the standard curve was greater than 0.98. 

### xCELLigence technology and application protocol

#### Technology

Two xCELLigence systems (ACEA Biosciences) were used throughout this study. The SP E96 well plate system and the DP 3x E16 well plate system [11]. See ACEA website for complete system specifications. The plates comprise a high-density gold electrode array covering approximately 80% of the surface area of the well. The electrode array directly measures changes in electrical impedance as a function of cell adhesion across the array. Each well operates and records its data independently. 

#### Cell-Index

Changes in electrical impedance are detected by the array and converted by the software (version 1.2) to Cell Index. In the simplest terms the higher the Cell Index value the greater the extent of cell adhesion. It is extremely important to note that xCELLigence only measures changes in adhesion of living adherent cells. Non-adherent or suspension cells do not influence the electrical impedance until adhesion begins. In principal, any cellular behaviour that acutely or substantially affects cell adhesion can be investigated using xCELLigence technology. We have successfully pioneered the use of xCELLigence technology to investigate the cytotoxic effect of human NK cells on astrocytes previously [11]. In the current study, we utilise the real-time dimension of the Cell-Index adhesion curves to monitor the differential astrocyte responsiveness to various pro-inflammatory cytokines. 

#### Protocol

In preliminary experiments, NT2-astrocytes were seeded at various sub-confluent cell densities to ascertain whether the cytokine response was influenced by the cell density. 

Cell density did not appear to have any influence on the observations (data not shown). NT2-astrocytes were seeded into E16 or E96 plates (Roche) at 6500 cells per well. Initially, 50 µl DF10 was placed into every well, ensuring the entire surface area is covered. Then the E16 or E96 plates were equilibrated to 37°C and then placed into the xCELLigence system to ascertain that all wells were recording within tolerances. Then cells were seeded in 50 µl of DF10 and allowed to settle for 10 minutes prior to starting recording. NT2-astrocytes are very adherent and do not require any matrix coatings, and will begin to adhere to plates within 2 minutes. 

### Quantification of nuclei

Following cytokine experiments the fixed astrocytes were stained with Hoechst and then images were acquired using a Leica DMR microscope mounted with a DFC425-C camera and Leica LASCore software. For each experiment at least 6 fields of view from at least 3 wells per treatment were acquired. Images were analysed using ImageJ software to automatically count the number of stained nuclei. In brief, images were converted to 8-bit and threshold levels set to reveal nuclei. The “analyse particles” function was used to count nuclei following optimisation of the Size and Circularity dimensions. 

### Statistical analysis

Statistical analysis was conducted by one-way ANOVA followed by Dunnett’s comparison (using GraphPad Prism) where the treatment group was compared directly to the control (vehicle). Statistical significance was achieved when *P < 0.05*. 

## Results

### GFAP expression in differentiated NT2 astrocyte cultures

**Figure 1:**
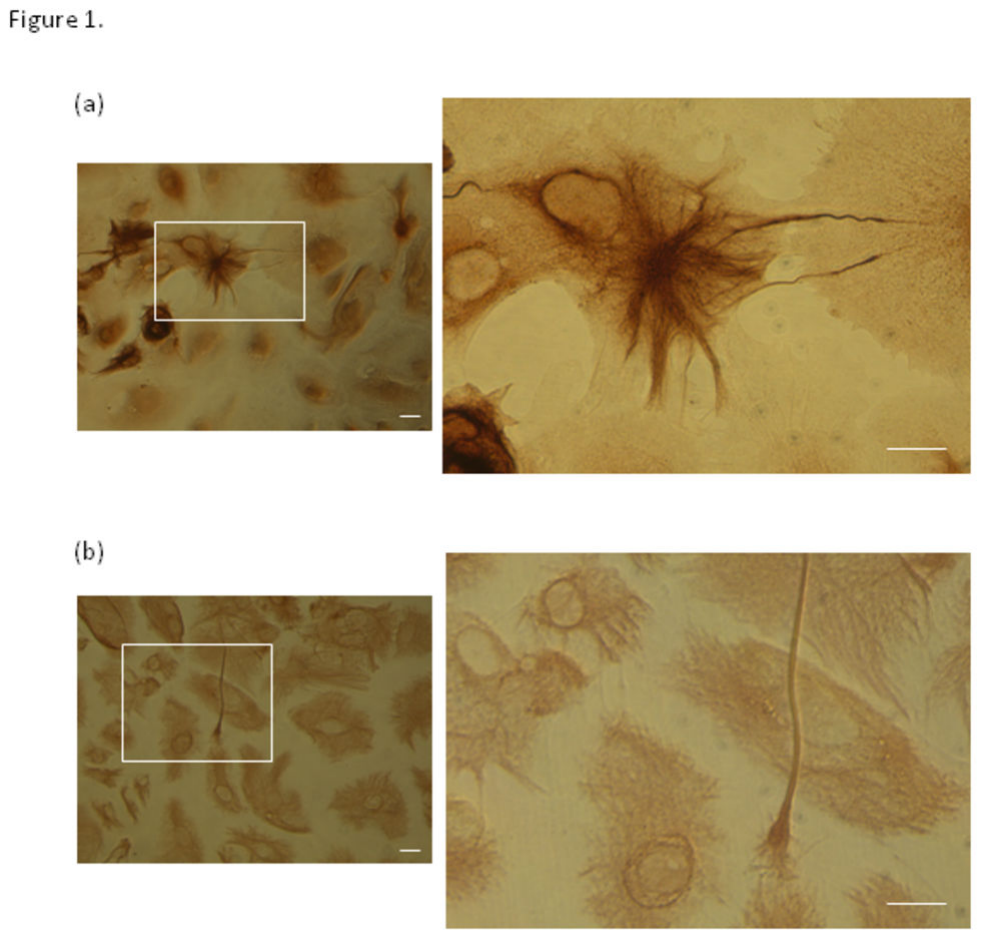
Expression of GFAP by NT2-astrocyte cultures. GFAP and vimentin expression in NT2 astrocytes. Images show the varied morphology of the (a) GFAP and (b) vimentin stained NT2 astrocytes. Most are strongly positive for GFAP staining, with some having highly pronounced intermediate-filament structures radiating from around centre of the cell body. Note that GFAP filaments are not present throughout the entirety of the astrocyte cytoplasm. The vimentin filaments are typically much finer are present throughout the astrocytic volume. Scale bars represents 20 µm.

The astrocytes used throughout this study were obtained by differentiation of the NT2 precursor cell line, which yields well characterised neuronal cells [14-17] and astrocytes [8,18]. The differentiation of the astrocytes takes 10-11 weeks, after which the harvested cells were routinely assessed for the expression of GFAP, vimentin and MAP 2. Freshly differentiated astrocyte cultures occasionally contain a few MAP 2 positive neuronal cells; however, these do not survive cryopreservation. The differentiated astrocytes are GFAP and vimentin positive (see Figure 1). Expression of GFAP shows considerably more variation in location and expression levels than vimentin. In addition, GFAP was poor at revealing the distal regions of the astrocytic cytoplasm. This is apparent in the right-hand panel of Figure 1a. The finer vimentin filaments reveal more of the astrocytic cytoplasm than GFAP. The expression of GFAP and vimentin by the NT2-astrocyte cultures reveals the highly variable and complex morphology of these cells, consistent with previous observations [19] and primary astrocytes [20,21]. 

### Time-Course of Inflammatory Cytokine Secretion of NT2 Astrocytes by TNFα and IL-1Β

In our previous study, we assess secretion of a large panel of inflammatory mediators from the differentiated NT2 astrocytes [8]. This was extended here to better understand the time-course of inflammatory activity and identify dynamic differences in response by the astrocytes. 

**Figure 2:**
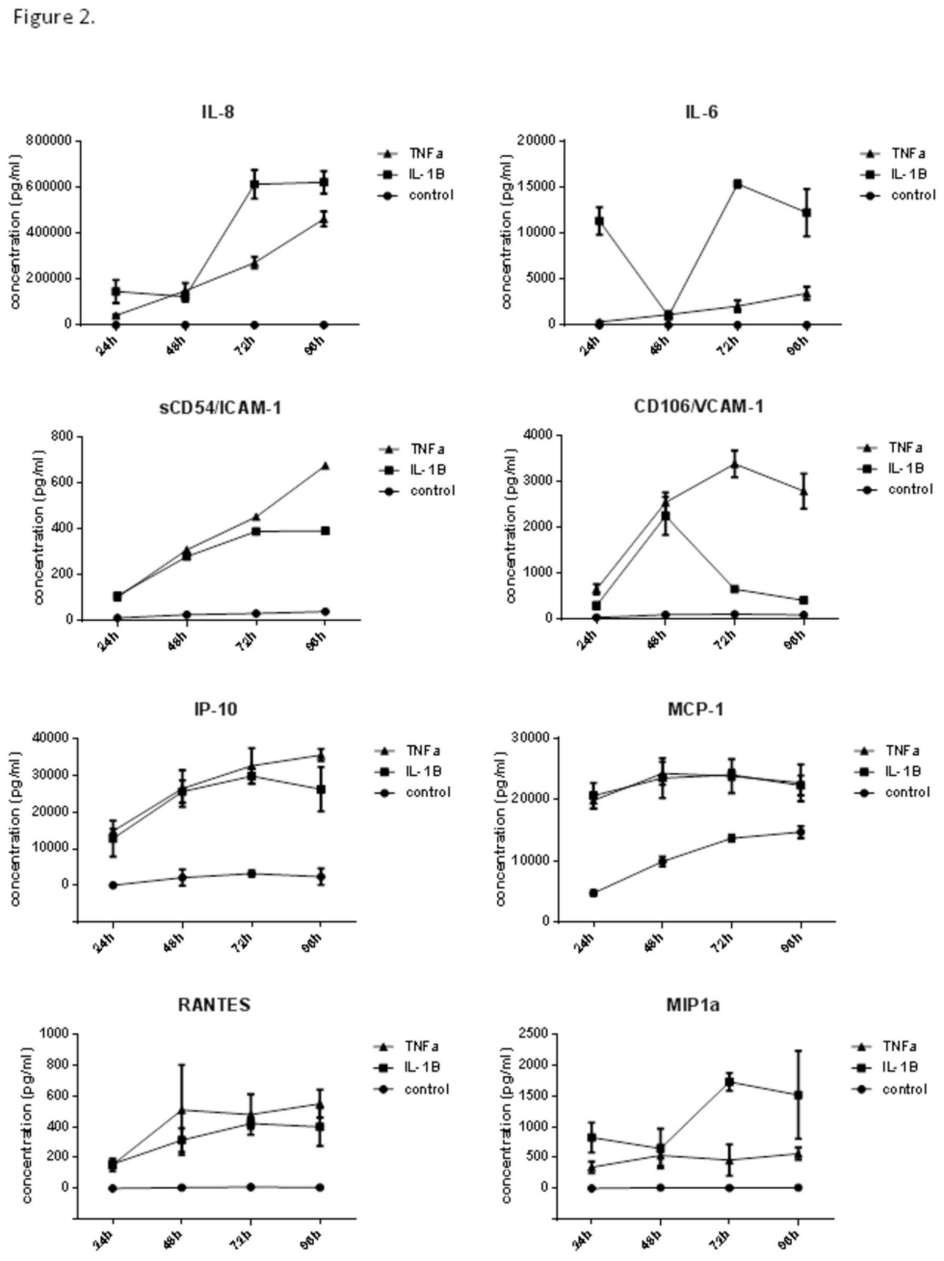
Time-course of astrocyte inflammatory response to IL-1β and TNFα (cytokine secretion). The time-course of cytokine secretion following IL-1β and TNFα treatment shows pronounced inflammatory activation within the initial 48 hour period. In addition, the time-course reveals differential secretion of IL-6, MIP1α, IL-8 and VCAM liberation. The concentration of cytokine was measured using multiplex cytometric bead array. The temporal nature of the data is very powerful especially for IL-6 and VCAM-1, which both show pronounced time-dependent dynamic reductions in concentration, which would not have been identified with a single time-point analysis. All concentrations are represented as pg/mL. Data show the mean +- SE (N=4).

**Figure 3:**
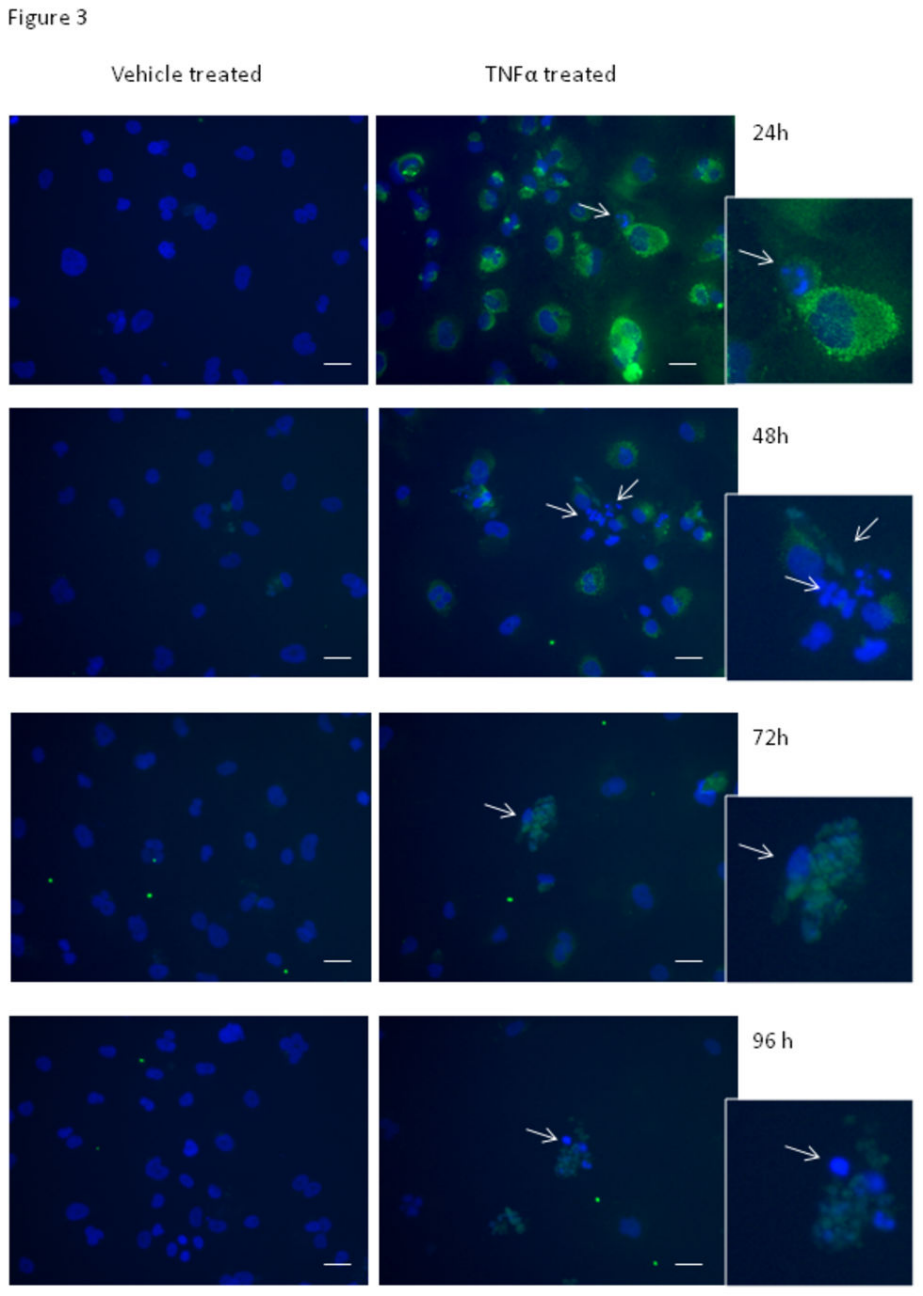
Analysis of intracellular expression of IP-10 following astrocyte activation. Induction of IP-10 and intracellular localisation following treatment with IL-1β or TNFα reveals a subpopulation of NT2-astrocyte which fail to produce IP-10 and have severely compromised nuclei. Cells were stimulated with either IL-1β (data not shown) or TNFα (5 ng/mL), or control media (vehicle) for 24 hours to 96 hours. IP-10 was absent in vehicle treated cells. Intracellular staining for IP-10 (green) shows elevated IP-10 levels 24 hours after cytokine addition in the majority of NT2-astrocytes (>95%). 48 hours after cytokine addition, there is a marked increase in the number of astrocytes with compromised nuclei (severely disintegrated or multi-lobed; abnormal). Detailed inspection reveals that these astrocytes have little or no IP-10 staining. In contrast, astrocytes with healthy looking nuclei typically have an abundance of chemokine present (especially at 24-48hrs). Note the smaller condensed nuclei and multi-lobed nuclei (highlighted by white arrows) present in the cytokine treated astrocytes. The response to IL-1β was very similar (data not shown). Scale bars are 40 µm.

Analysis of the cytokine production was conducted using a combination of cytometric bead array (Figure 2) and intracellular staining for specific chemokines that were produced at high levels by the astrocytes (Figure 3). A panel of key pro-inflammatory cytokines encompassing danger signals (IL-6, IL-8), potent chemokines (IL-8, MCP-1, IP-10, RANTES, and MIP1α) and leukocyte adhesion molecules (ICAM-1 and VCAM; leukocyte trafficking) were investigated. Samples were collected across a time-course of 96 hours to detect early and late responses and cytokine concentrations were measured in conditioned media using multiplexed cytometric bead array. This technology enables simultaneous measurement of each cytokine and has a lower detection limit of 1-10pg/mL. The only cytokine detected under basal culture conditions was MCP-1, which was secreted in a constitutive manner (Figure 2). Secretion of each cytokine was elevated in response to TNFα or IL-1β, with most elevated within 24 hours. The secretion of several of the chemokines (IP-10, MCP-1 and RANTES) peaked by 48 hours. This demonstrated that most of the inflammatory response occurred within the initial 48-hour period. The secretion profile in response to TNFα or IL-1β was similar for some of the cytokines measured, however, there were notable difference in the secretion of IL-6 (general danger signal) and sVCAM-1 (soluble VCAM; liberated from cell surface). The profile of IL-6 secretion induced by IL-1β was bimodal, with a large elevation within 24h. However, by 48 hours the IL-6 present at 24 hours had been cleared by the astrocytes. This was followed by a second substantial wave of IL-6 secretion, which only occurred with IL-1β treatment. Closer inspection of the IL-8 and MIP1α secretion reveals a similar profile to that of IL-6 (Figure 2). The detection of soluble ICAM-1 and VCAM in conditioned media represents liberation (shedding) of these membrane proteins by the astrocytes. There is a clear distinction in the amount of VCAM present in the condition media, which surprisingly declines after 48 hours following IL-1β treatment. This suggests that the cells have actively assimilated or cleared the soluble VCAM that had been released by the cells. These temporal data show that that although IL-1β and TNFα both potently activate the NT2 astrocytes, the inflammatory activity is temporally and dynamically quite different. 

**Figure 4:**
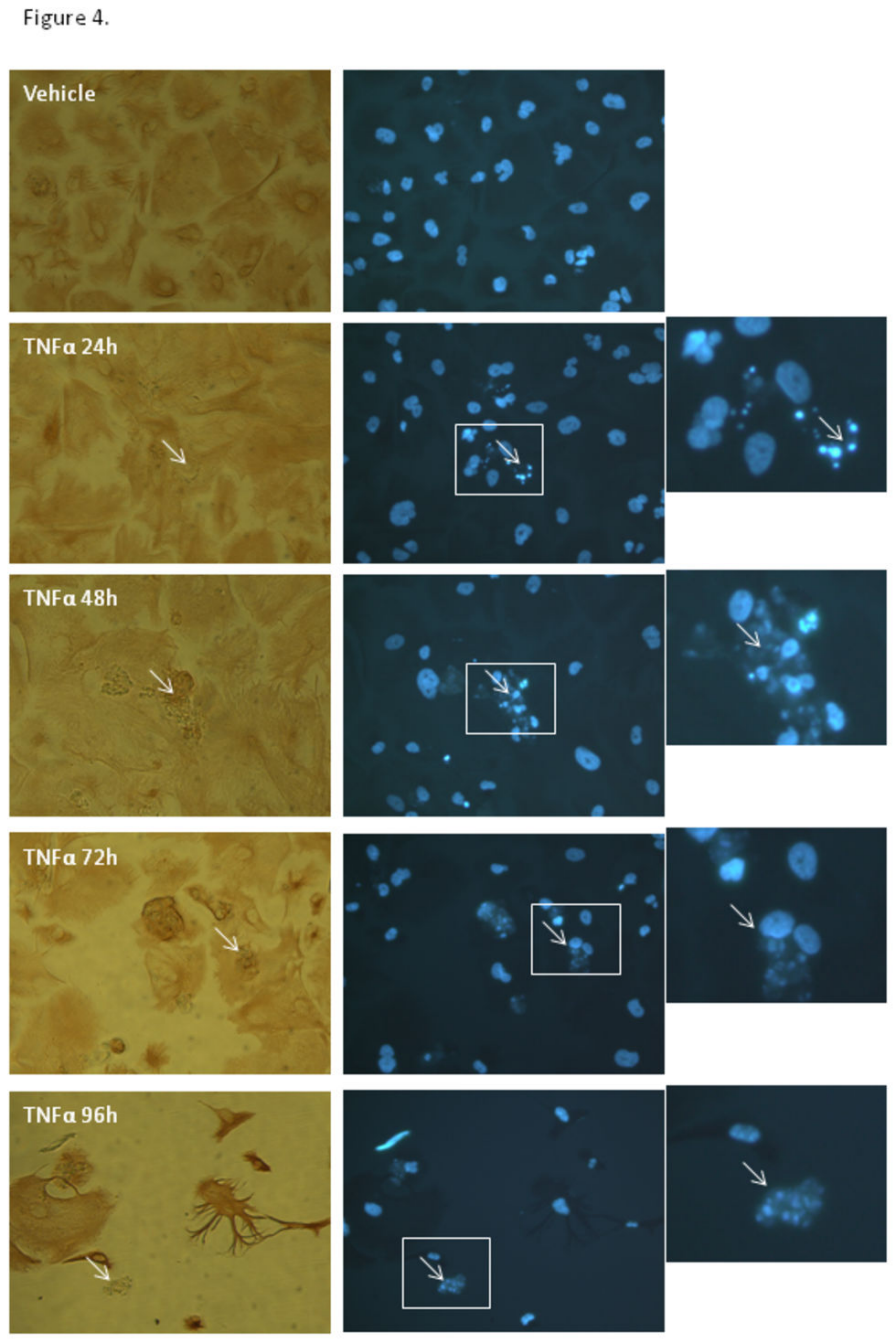
TNFα and IL-1β activation of astrocytes induces nuclear disintegration and cell compromise. Analysis of nuclear and cellular morphology further corroborates the earlier observation that activation with TNFα or IL-1β induces astrocyte compromise. Cells were stained with Hoechst and vimentin to reveal integrity of the nucleus and cytoplasm, respectively. The white arrow points to the same compromised cells for comparison between the vimentin and nuclear staining. The enlarged region of interest within the white box shows an example of a highly compromised astrocyte with a severely disintegrated nucleus. Data are shown for TNFα treatment. Note also the loss of following cytokine treatment of the astrocytes with time (72 and 96 hours).

Intracellular staining for several cytokines was conducted, including MCP-1 (data not shown) and IP-10 (Figure 3). 24h after treatment >95% of the NT2 astrocytes were positive for IP-10. The intracellular localisation of IP-10 progressively diminishes from 48h onwards. In addition, there were notable numbers of compromised astrocytes 48h after stimulation. The compromised astrocytes had abnormal nuclei (see Figure S1), lacked chemokine staining and often had a swollen appearance. This was even more evident 72 hours after treatment with TNFα or IL-1β (IL-1β data not shown), where there was also a noticeable loss of cells (Figure 3 and Figure 4). The white arrows point out good examples of compromised astrocytes. Figure 4, shows the morphological changes (DAB stained images) and nuclear disintegration following TNFα activation (data for IL1β was similar; not shown). Nuclear compromise was evident as early as 24 hours. The white boxed regions highlight good examples of highly compromised astrocytes, which were not observed in the vehicle treated cells. The white arrows point to the same cell for comparison of the vimentin staining and nuclear compromise. Inspection of the vimentin staining reveals that the highlighted cell is highly compromised and lacks the normal morphology of the control treated astrocytes. 

The astrocytes remaining at 96 hours post-stimulation have a greatly altered morphology (see Figure 4 and Figure S2). Often the cytoplasm is greatly shrunken or highly branched as shown in Figure 4. The most striking observation is the substantial cell loss at 96 hours post cytokine treatment. Collectively, these data show that the NT2 astrocytes are dynamically responsive to TNFα and IL-1β, which results in the pronounced inflammatory activation of the astrocytes prior to progressive compromise and death. 

Abnormal apoptotic nuclei are present as early as 24 hours in vimentin positive (Figure 3) and GFAP positive astrocytes (Figure S1). These astrocytes are clearly unhealthy and usually have very shrunken cell bodies. 

### Astrocyte adhesion is compromised by chronic treatment with TNFα and IL-1β, but not by other pro-inflammatory mediators

**Figure 5:**
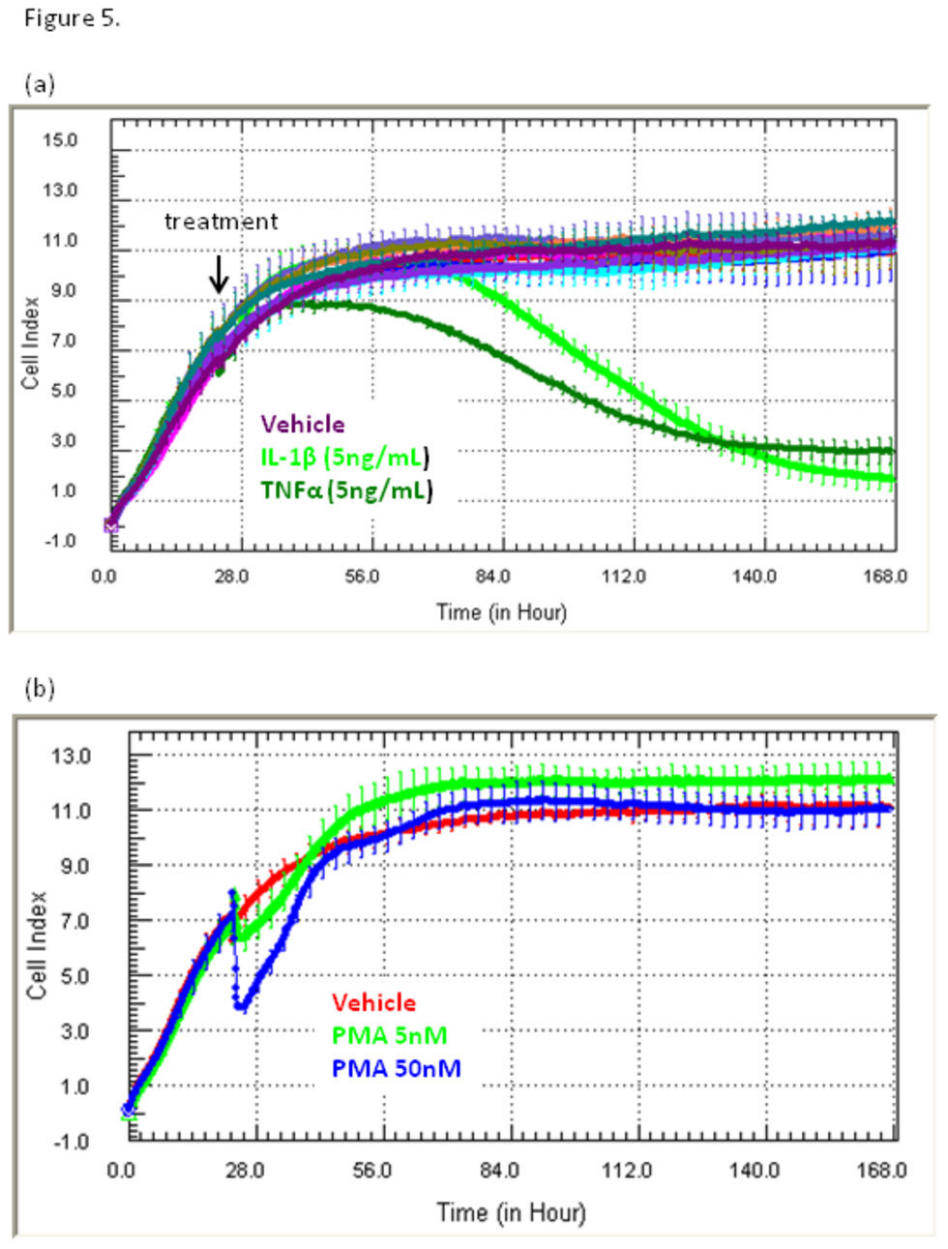
Astrocyte adhesion is compromised by treatment with TNFα and IL-1β, but not by other pro-inflammatory mediators. NT2-astrocytes were treated with a range of pro-inflammatory mediators including IL-1β and TNFα (5 ng/mL) 24 hours after seeding into E96 xCELLigence biosensor plates (indicated by black arrow). The xCELLigence biosensor measures net adhesion (Cell Index) of living cells. (a) The individual profiles for IL-1β (green) and TNFα (dark green) reveal that total adhesion levels decline progressively with time. On set of loss usually occurs within the first 24-48 hours after stimulation with IL-1β or TNFα. This does not occur in cells treated with the following chemokines (all used at 50ng mL); SDF-1 (red), IL-8 (aqua), IP-10 (brown), MCP-1 (tan), MIP1α (blue), MIP1β (pink), RANTES (orange) or control media (purple). Furthermore, the cytokines IFNγ, IL-2 and IL-4 had no effect on astrocyte survival (curves not shown). The lower panel (b) shows the response to PMA (5nM and 50nM), where there is an immediate transient reduction in adhesion (Cell Index). This transient reduction rebounds back to the vehicle Cell Index values, indicating that the response is acute, but is not cytotoxic. The plotted data shows the mean +- SD (n=6 wells from a representative experiment). The progressive loss of adhesion following IL-1β and TNFα has been observed in more than 20 independent xCELLigence experiments.

The time-course of astrocyte responses to the inflammatory cytokines TNFα and IL-1β was further investigated using xCELLigence biosensor technology (Figure 5), which measures net cellular adhesion (see methods section for full description of this technology) in real-time. The xCELLigence data clearly reveals that TNFα and IL-1β treatment results in substantial time-dependent loss of astrocyte adhesion (Figure 5a). Loss of adhesion occurred progressively over a 3 to 5 day period after addition of cytokine. This progressive loss in adhesion was greater than 60% in all experiments. Progressive and permanent loss of adhesion was observed in all experiments. This is consistent with compromise and death of cells reported in other xCELLigence studies [22,23]. 

The effects of IL-1β and TNFα were also tested on the NT2 precursor cells (undifferentiated) and pure neurons (see Figure S3). However, neither of these cell types responded and the Cell Index curves were very different for both cell types in comparison to the astrocyte curves (Figure S3). Along with the GFAP expression (Figure 1), this further validated that the cytokine responses were from the astrocytes. 

IL-1β and TNFα are often released in the CNS during neuroinflammation [3,24,25]. However, numerous other inflammatory factors have been detected in the CNS during neuroinflammation. We therefore assessed a range of cytokines (interferon, interleukins and chemokines) with known importance to CNS inflammation [26-28] to see if they induced similar effect to IL-1β and TNFα. The pro-inflammatory mediator IFNγ did not affect astrocyte adhesion, nor did IL-2, IL-4 or GMCSF, which were used as negative controls (Figure 5a). We also assessed responses to several chemokines, the receptors of which, have been shown by others to be expressed by human astrocytes [4,6]. This included SDF-1 (CXCL12; ligand of CXCR4), IL-8 (CXCL8; ligand for CXCR1 and 2) and IP10 (CXCL10; ligand for CXCR3); none of these CXCL chemokines affected astrocyte adhesion or survival. We also investigated the effects of several CCL chemokines, each reported to be associated with CNS inflammation [29-31]. This included MCP-1 (CCL2), MIP1α (CCL3), MIP1β (CCL4) and RANTES (CCL5); however, none had any effect on astrocyte compromise (Figure 5a). In addition, the synthetic agonists of PKC (PMA) and adenyl cyclase (forskolin) were tested across a variety of concentrations. Although PMA increased secretion of a number of inflammatory cytokines similar to IL-1β and TNFα [8], PMA did not induce astrocyte compromise and death (Figure 5b). PMA induced a transient reduction in astrocyte adhesion, but did not have any long-term detrimental effects on adhesion. No morphological changes (blebbing, lysis or cell loss) were evident in astrocyte phenotype following PMA treatment (data not shown). Forskolin had no effect (data not shown), consistent with the lack of effect on cytokine production [8]. This demonstrated that loss of adhesion and compromise induce by IL-1β and TNFα, was not reproduce by a range of other inflammatory mediators found in the CNS. 

### Compromise of astrocyte adhesion induced by TNFα and IL-1β occurs in a concentration-dependent manner

**Figure 6:**
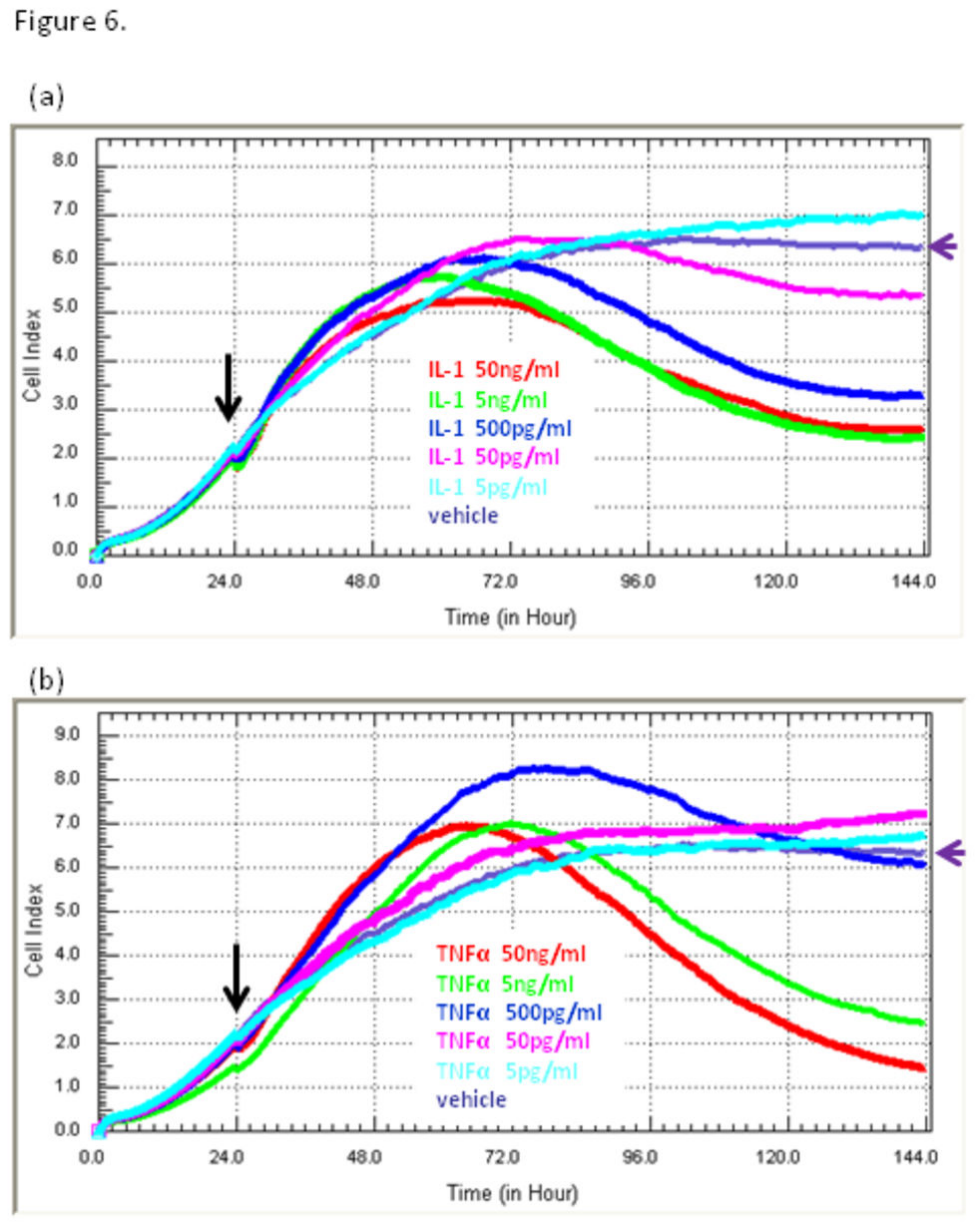
Compromise of astrocyte adhesion induced by TNFα and IL-1β occurs in a concentration-dependent manner. NT2-astrocytes were stimulated with a range of concentrations of IL-1β and TNFα (50 ng/mL to 5pg/mL) 24 hours after seeding into E96 plates (indicated by black arrow). The individual pharmacological profiles for (a) IL-1β and (b) TNFα reveal that the on-set of compromise (decline in adhesion levels, Cell Index) is concentration dependent. The cytokine concentrations are colour-coded to highlight the subtle difference in the responsiveness to IL-1β or TNFα. xCELLigence revealed that the responses were similar but not identical. The black arrow signifies addition of cytokines and the purple arrow labels the control treated cells for reference (purple curve). Each curve is the mean of 6 treatments. The SD bars have been omitted for simplification of the figure.

The sensitivity of the NT2 astrocytes to TNFα and IL-1β was investigated across a concentration range of 50 ng/mL to 5 pg/mL. Typically, the astrocytes were responsive to TNFα and IL-1β at concentrations above 50 pg/mL. Following addition of IL-1β (Figure 6(a)) or TNFα (Figure 6(b), (50 ng/mL to 5pg/mL)) there is a pronounced transient increase in total adhesion (Cell Index increase) in the subsequent 24-48 hour period. This transient increase was only observed in some experiment. We hypothesis this is related to increased inflammatory activation of the astrocytes (e.g. increased adhesion molecule expression or increased astrocyte volume). After this period of activation, where we know that production of numerous inflammatory cytokines increases [8] (see Figure 2), there is a progressive decline in Cell Index. The permanent loss of adhesion occurred in all experiments. This reduction in adhesion occurs relatively slowly (progressive rather than rapid), requiring at least 72 hours to reach the minimum Cell Index levels attained (figure 5 and 6). Even at the highest concentration used (50 ng/mL) the Cell Index values do not reach zero. This indicates that not all of the NT2 astrocytes are sensitive to the same extent. 

### Progressive loss of adhesion correlates with astrocyte compromise

**Figure 7:**
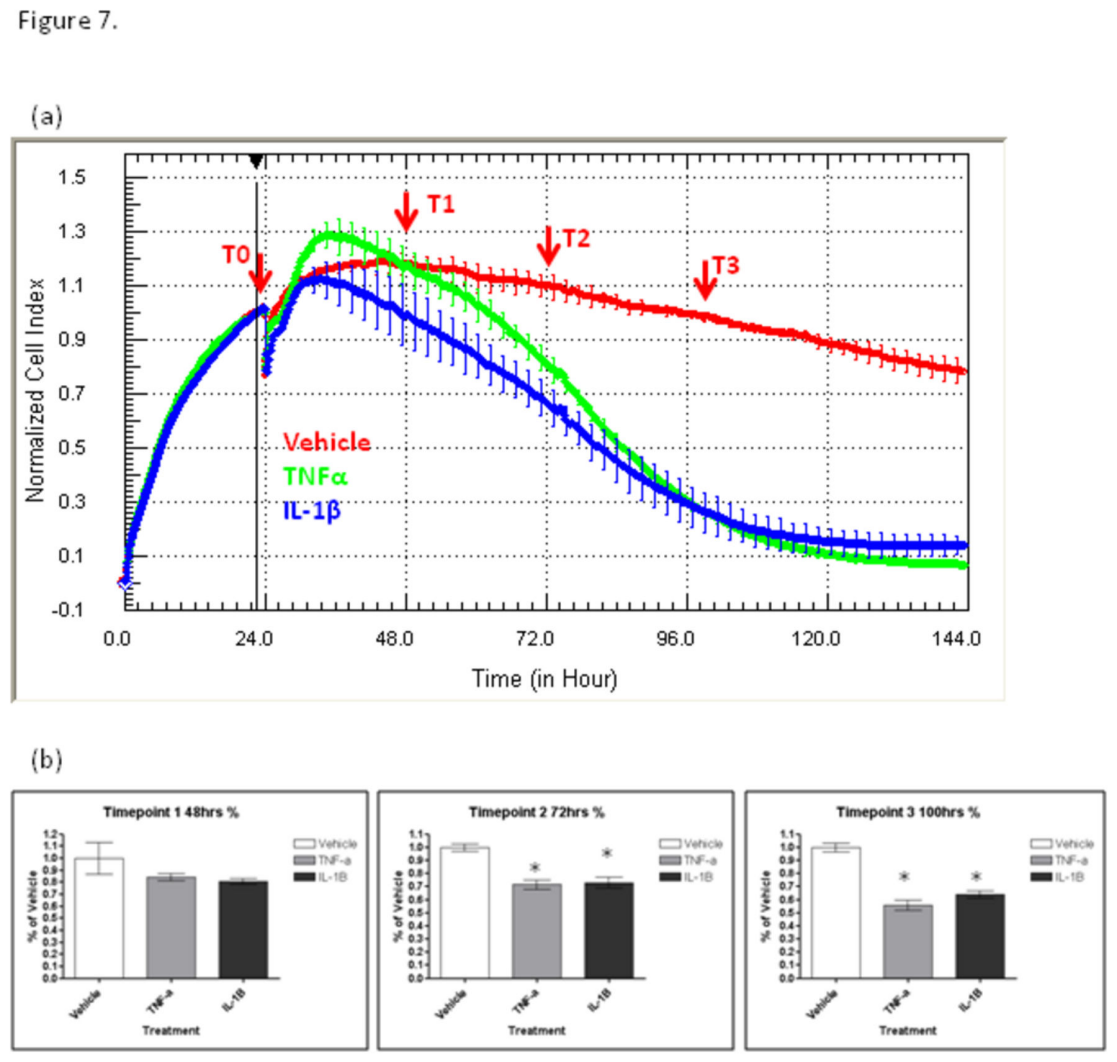
Progressive loss of adhesion correlates with astrocyte compromise. NT2 astrocytes were prepared in parallel for quantification of ATP levels (mitochondrial function) and measurement of loss-of-adhesion with xCELLigence. The response to IL-1β and TNFα (5 ng/mL) was monitored in real-time to identify the time-points for conducting the ATP assays, which were conducted using parallel plates. Cytokines were added at T0. The time point 1 (T1) represents the early loss of adhesion, T2 represents significant reduction in Cell Index (i.e. reduction in astrocyte adhesion), T3 represents major sustained reduction in Cell Index. (b) quantification of astrocytes ATP levels using parallel plates terminated at the indicated time-points (T1 to T3). Significance level is P < 0.05. Data are from a single experiment, which is representative of 4 independent experiments.

Next, the loss of adhesion was investigated to ascertain when this response translated into a significant difference in cellular ATP levels, which was used as a measure of cell viability (mitochondrial function). Astrocytes were set up in 96-well plates (ATP assay) and xCELLigence plates in parallel for precisely pinpointing the initial loss of adhesion. Figure 7a shows the xCELLigence data highlighting the time-points when the endpoint ATP assays were conducted. The shape of the Cell Index curves revealed that loss-of-adhesion had commenced at the first time-point (T1), which was ~24 hours post-cytokine addition. However, at the first time-point the reduction in ATP levels were not significantly different statistically. Cell adhesion continues to decline with time and the ATP levels measured 48 hours after cytokine treatment (T2) are significantly lower than the vehicle treated astrocytes (P < 0.05). From 48 hours onwards, the loss of adhesion and reduction in intracellular ATP levels (Figure 7b), suggest that the early loss-of-adhesion measured by xCELLigence is a function of astrocyte compromise. 

### IL-1β and TNFα induce nuclear condensation, cleaved caspase-3 expression and death of NT2-astrocytes

The xCELLigence real-time readout is powerful for investigating specific events in real-time. This parameter was utilised here to compare loss of adhesion (compromise) with expression of cleaved caspase-3 (dying) and loss of cells (death). 

The quantification of cell-loss was conducted by counting the number of remaining nuclei. Our software could not differentiate between healthy and compromised nuclei (see figures 3 and 4). Therefore, the data shows how many cells are remaining however, it should be noted that at each time point that compromised nuclei are present, therefore the actual number of healthy cells at each time-point following cytokine treatment is lower. From 48 hours onwards, astrocyte numbers were reduced, but this reduction did not reach significance statistically at 48 hours. The quantification of cell counts was comparable to the quantification of cellular ATP (Figure 7). 

**Figure 8:**
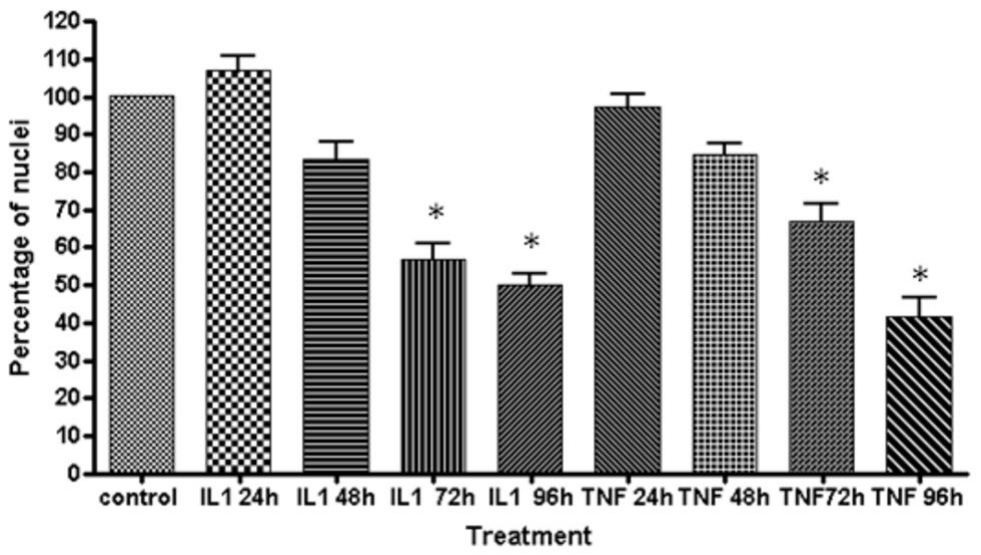
IL-1β and TNFα induced astrocyte death. Astrocytes were stimulated across a 96-hour time course to assess the extent of cell loss following IL-1β and TNFα treatment. Cell numbers were quantified by counting Hoechst stained nuclei. However, healthy and compromised nuclei were not discriminated by the automated software used. Data are from a single experiment quantified from 6 fields of view. Data are representative of three independent experiments. Significance where P < 0.05* .

**Figure 9:**
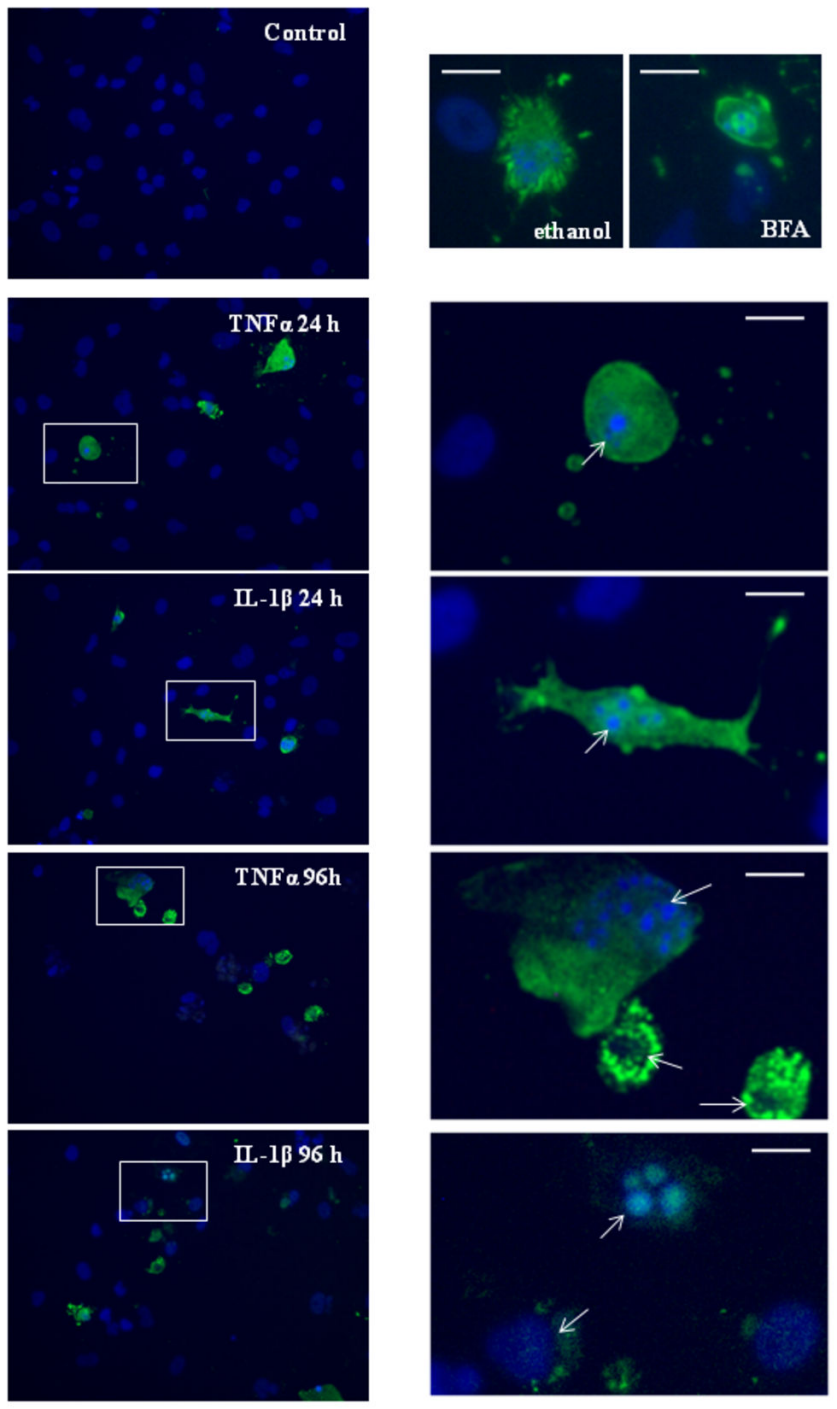
Induction of cleaved caspase-3 and nuclear disintegration following IL-1β and TNFα treatment. Expression of cleaved caspase 3 was investigated to assess whether it was present during the early stages of cytokine-induced compromise. Panels show cleaved caspase 3 expression in green and nuclei in blue. The white boxes highlight cleaved caspase 3 positive astrocytes, with the enlarged panel showing the severe disintegration of the nucleus. In many cases, the nucleus was visible only as a small (<5 µm) intense vesicle or series of vesicle like structures, consistent with apoptotic nuclei. Cleaved caspase 3 was not detected in vehicle treated cells. Ethanol (1%) and Brefeldin A (BFA 10 µM) were used as positive controls [46,47]. The scale bar represents 20 µm for reference. Data representative of 4 independent experiments.

Both IL-1β and TNFα induced expression of cleaved caspase-3 at all time-points investigated (24 hours to 96 hours after cytokine addition). Even though there was no significant cell loss 24 hours after cytokine treatment (Figure 8), there was expression of cleaved caspase-3 within 24 hours of IL-1β and TNFα treatment (Figure 9) and this correlated with compromised nuclei (Figure 9). This was consistent with the expression of cleaved caspase-3 and apoptotic nuclei induced by the positive controls (see Figure 9 and Figure S4). In all of the cleaved caspase-3 positive astrocytes, the nuclei were severely disintegrated, often present in numerous condensed vesicles-like structures (see Figure 9). In other cleaved caspase 3 positive astrocytes, the nuclear DNA stained very weakly with Hoechst (which requires double stranded DNA), thus giving the appearance of the cell not actually having a nucleus. For these two reasons, quantification of cleaved caspase-3 positive cells proved too unreliable. None-the-less, cleaved caspase 3 expression was present from 24 hours onwards after cytokine treatment, with the majority of astrocytes present beyond 96 hours expressing cleaved caspase 3. In contrast, cleaved caspase 3 was never detected in vehicle treated cells. 

This demonstrates that the early loss of adhesion is an early marker of the nuclear compromise and apoptosis events that follow. The expression of cleaved caspase-3 and disintegrated nuclei within 24 hours demonstrates that some astrocytes were already committed to an apoptotic fate within this time-frame. 

## Discussion

Astrocytes play an essential role in the central nervous system in both health and disease [32]. It is now very clear that astrocytes represent a complex heterogeneous and functionally diverse population of cells [1]. Exemplars of this are the astrocytes that surround neuronal synapses [33] (e.g. glutamatergic synapses) in the human hippocampus and astrocytes that are associated with the blood brain barrier (BBB) [20,34]. Not all astrocytes express the same proteins or cell surface receptors, or respond to the same growth factors or inflammatory cytokines [1]. In the context of neuroinflammation, especially where there is activation or damage to the BBB, astrocytes associated with the neurovascular unit are believed to have key functions involved with maintaining BBB integrity. These astrocytes are a source of the parenchymal basement membrane, and produce a variety of growth factors to maintain the unit (for an eloquent review see; [2]). It is relatively easy to envisage how dysfunction of cells integral to the BBB could cause or exacerbate brain injury. 

We are particularly interested in the role astrocytes play during brain injury in states of neuroinflammation. They represent a target to protect, in a number of different neurological conditions. This includes conditions where astrocyte compromise exacerbates brain injury or where astrocyte loss may reduce tissue integrity (BBB) or neuronal support. This can occur during the inflammatory response where the inflammatory milieu is cytotoxic to surrounding viable cells (e.g. resulting in death of neurons) or indirectly as a consequence of glial cell loss, which will impact on the reparative mechanisms and also on neuronal homeostasis. Here we have investigated the longer-term effects of various inflammatory cytokines including IL-1β, and TNFα, which are widely implicated in the immunological regulation of astrocyte biology and CNS immunology [4,32,35]. 

Previously we found that NT2-astrocytes secrete a number of neutrophil and monocyte chemokines in response to TNFα and IL-1β [8]. We have advanced our earlier study by investigating the temporal inflammatory astrocytic response using cytometric bead array and intracellular chemokine staining. An important finding of the temporal analysis of cytokine/chemokine secretion is that it reveals that the inflammatory output in response to following TNFα is different to the response to IL-1β treatment. This is consistent with the gene array studies showing cytokine specific responses by human primary astrocyte [6]. This is further validation of the usefulness of the NT2-astrocytes, especially in the absence of human brain cultures enriched for astrocytes. As primary astrocytes are phenotypically heterogeneous, we also investigated the intracellular chemokine (IP-10 and MCP-1) expression to assess the heterogeneity of the NT2-astrocyte response. The vast majority of NT2-astrocytes increased intracellular chemokine levels, indicative of a homogeneous response to TNFα and IL-1β. However, on close inspection there were a few astrocytes that completely lacked any chemokine staining. These astrocytes had abnormal appearing nuclei, which were a mixture of multi-lobed (disintegrated) or highly condensed nuclei. The abnormal nuclear morphology combined with the lack of chemokine staining suggested that the cytokines, IL-1β and TNFα, induced astrocyte compromise, thus impairing production of the inflammatory chemokines. Importantly, this compromise was evident as early as 24 hours following cytokine treatment. 

Although xCELLigence technology is relatively new, there are over 150 publications to date validating its use for the measurement of numerous cellular responses. One of the best applications for xCELLigence is the measurement of cell compromise and subsequent cell death [13,22,36-38]. xCELLigence measures cell adhesion (represented as Cell-Index) and loss of adhesion occurs early during membrane compromise and cell death. The permanent loss of adhesion (as measured by xCELLigence) signifies irreparable compromise. The xCELLigence data clearly shows that the loss of adhesion was progressive (gradual), typically across 24-96 hours (and longer in some experiments) after cytokine treatment. One of the advantages of xCELLigence is that the data output is in real-time. This therefore was used to temporally compare loss of adhesion with cellular compromise (APT levels/nuclear disintegration), expression of cleaved caspase-3 and finally cell loss. Overall, as demonstrated between figure 3-9, this comparison revealed that loss of adhesion occurred prior to actual cell loss but coincided with expression of cleaved caspase-3 and nuclear disintegration. Thus, the xCELLigence biosensor was valuable at revealing the early stages of astrocyte compromise, which was followed by cell death (apoptosis) in a time-dependent manner. 

Contrary to the cytotoxic effects of IL-1β and TNFα, various other mediators of CNS inflammation were not cytotoxic to astrocytes. This included the CXCL chemokines SDF-1, IL-8 and IP-10 and the CCL chemokines MCP-1, MIP1α, MIP1β and RANTES. Furthermore, the cytokines IFNγ, IL-2 and IL-4 had no effect on astrocyte survival. For these cytokines and chemokines, there was neither an increase nor decrease in astrocyte adhesion levels, which suggested that any effects on the NT2-astrocytes adhesion characteristics were minimal. In addition, the synthetic agonist of PKC signalling, PMA, which activates astrocytes and increases production of a variety of inflammatory pathways and chemokines, did not have any cytotoxic effects we could detect using xCELLigence technology. This was an interesting observation as PMA increased production of most of the cytokines induced by IL-1β and TNFα, but without the secondary cytotoxic effect [8]. 

We have revealed that cleaved caspase-3 is induced in NT2-astrocytes by TNFα and IL-1β. Importantly, this pro-apoptotic factor is detectable within 24 hours of cytokine treatment (TNFα and IL-1β), but was not observed in vehicle treated cells. However, we also observed compromised astrocytes with severely disintegrated multi-lobed nuclei that lacked cleaved strong caspase-3 expression (examples are present in Figure 9). Based on the morphology of these cells its highly unlikely they were viable, but we can not conclude the involvement of cleaved caspase 3 in their demise. In addition, cleaved caspase-3 positive astrocytes were present, where the nucleus was almost invisible. This suggested that the nuclear disintegration was very advanced and that the remnants were likely dead. 

Numerous inflammatory cytokines are elevated in the CNS following injury [39-41]. In various neurological conditions elevated serum or CSF levels of specific cytokines correlate with poor neurological outcomes [42-44]. These include TNFα and IL-1β, which have been shown to impair blood-brain barrier function [39,45]. We show that secondary to inflammatory activation of astrocytes by TNFα and IL-1β [8], that the longer-term effect of these cytokines is detrimental to the survival of astrocytes. This reveals a new potential cellular target, which may help explain some of the adverse tissue damaging effects of these cytokines that occur during neuroinflammation. 

## Supporting Information

Figure S1 **Nuclear compromise evident in GFAP positive astrocytes 24 hours treatment with IL-1β and TNFα.** GFAP expression is indicated by the brown (DAB) precipitate, where as nuclei are stained with Hoechst (blue). The white arrows point to highly compromised astrocytes, with severely shrunken cell bodies and apoptotic nuclei. Scale bar is 25 µm.
(TIF)

Figure S2 **Nuclear compromise and loss of astrocyte morphology following chronic treatment with IL-1β and TNFα.** 4 to 5 days after exposure to the inflammatory cytokines (IL-1β and TNFα; 5 ng/mL), the vimentin intermediate filament staining (brown DAB signal) reveals an altered astrocytic morphology. Most of the living astrocytes are considerably shrunken, consistent with the reduction in adhesion (xCELLigence data) and there are remnants of several cells highlighted by the arrows that have fragmented nuclei (process referred to as karyorrhexis) severely degraded nuclei or lack vimentin staining. The bright field images reveal that these cells are dead. We also observed astrocytes with an active fibrous morphology, which we have observed phagocytosing dead cells (debris) using time lapse microscopy (data not show). The astrocyte in the 120hr TNFα panel is in contact with the remnant of a dead astrocyte, possibly in the process of engulfing the debris. The treatment and duration are detailed in the panel. Scale bars are 50 µm.
(TIF)

Figure S3 **Analysis of NT2 precursor and neuronal adhesion with xCELLigence.** Treatment of the (a) NT2 precursor cells or the (b) differentiated neuronal cells with either TNFα or IL-1β does not result in any loss in adhesion (no change in Cell Index), which is in contrast to the response from the astrocytes. The profile of the precursors is consistent with proliferating cells, whereas the neuronal cells produce a very low Cell Index adhesion level. These Cell Index curves are substantially different to the astrocyte curves and show no response to cytokine treatment. The arrows shows when cytokine treatments were added.
(TIF)

Figure S4 **Induction of cleaved caspase 3 by ethanol and Brefeldin A.** Ethanol (1%) and Brefeldin A (BFA; 10 µM) were used as positive controls to induce cleaved caspase 3 expression in the astrocytes. The white boxed area has been enlarged to highlight the destruction of the nucleus, which has been reduced to numerous small vesicle like structures. This pattern of nuclear damage and cleaved caspase 3 was identical to that induced by IL1β and TNFα. Scale bar is 50 µm.
(TIF)
