# Supplementary figures and images for: Exposure to Inflammatory Cytokines IL-1β and TNFα Induces Compromise and Death of Astrocytes; Implications for Chronic Neuroinflammation
Source: PLoS One. 2013 Dec 19;8(12):e84269. doi: 10.1371/journal.pone.0084269 (PMC3868583; doi:10.1371/journal.pone.0084269)

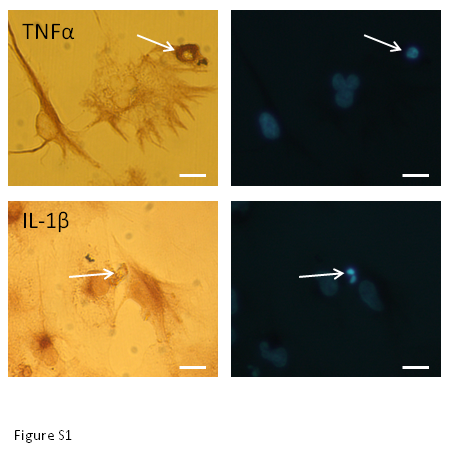

Supplement: Figure S1 — Nuclear compromise evident in GFAP positive astrocytes 24 hours treatment with IL-1β and TNFα. GFAP expression is indicated by the brown (DAB) precipitate, where as nuclei are stained with Hoechst (blue). The white arrows point to highly compromised astrocytes, with severely shrunken cell bodies and apoptotic nuclei. Scale bar is 25 µm. (TIF) [file pone.0084269.s001.tif]

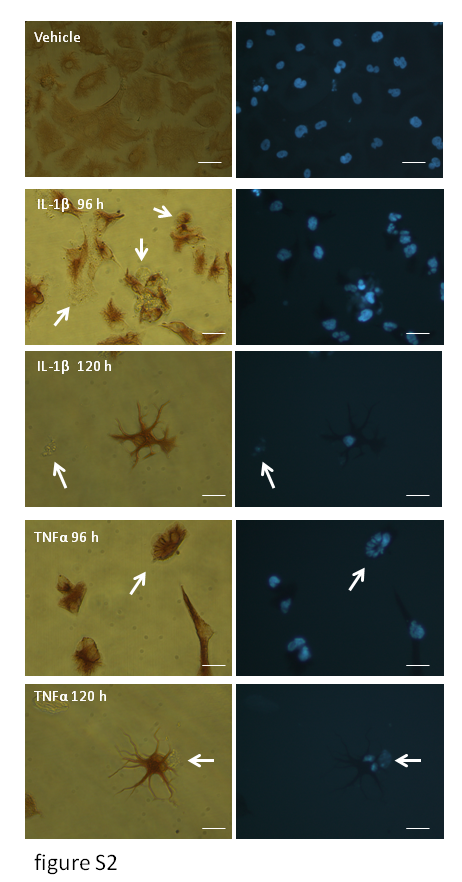

Supplement: Figure S2 — Nuclear compromise and loss of astrocyte morphology following chronic treatment with IL-1β and TNFα. 4 to 5 days after exposure to the inflammatory cytokines (IL-1β and TNFα; 5 ng/mL), the vimentin intermediate filament staining (brown DAB signal) reveals an altered astrocytic morphology. Most of the living astrocytes are considerably shrunken, consistent with the reduction in adhesion (xCELLigence data) and there are remnants of several cells highlighted by the arrows that have fragmented nuclei (process referred to as karyorrhexis) severely degraded nuclei or lack vimentin staining. The bright field images reveal that these cells are dead. We also observed astrocytes with an active fibrous morphology, which we have observed phagocytosing dead cells (debris) using time lapse microscopy (data not show). The astrocyte in the 120hr TNFα panel is in contact with the remnant of a dead astrocyte, possibly in the process of engulfing the debris. The treatment and duration are detailed in the panel. Scale bars are 50 µm. (TIF) [file pone.0084269.s002.tif]

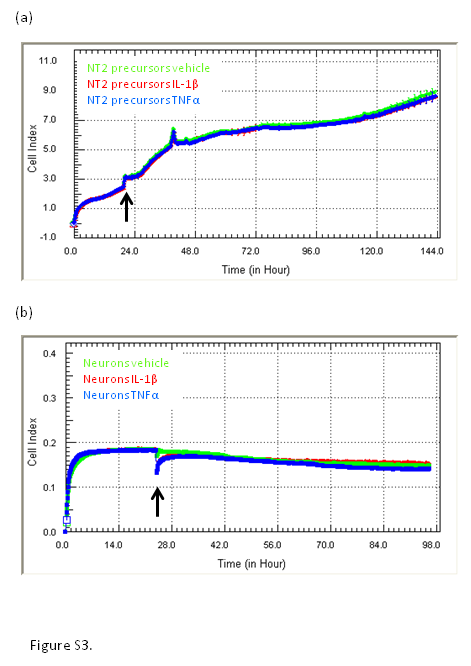

Supplement: Figure S3 — Analysis of NT2 precursor and neuronal adhesion with xCELLigence. Treatment of the (a) NT2 precursor cells or the (b) differentiated neuronal cells with either TNFα or IL-1β does not result in any loss in adhesion (no change in Cell Index), which is in contrast to the response from the astrocytes. The profile of the precursors is consistent with proliferating cells, whereas the neuronal cells produce a very low Cell Index adhesion level. These Cell Index curves are substantially different to the astrocyte curves and show no response to cytokine treatment. The arrows shows when cytokine treatments were added. (TIF) [file pone.0084269.s003.tif]

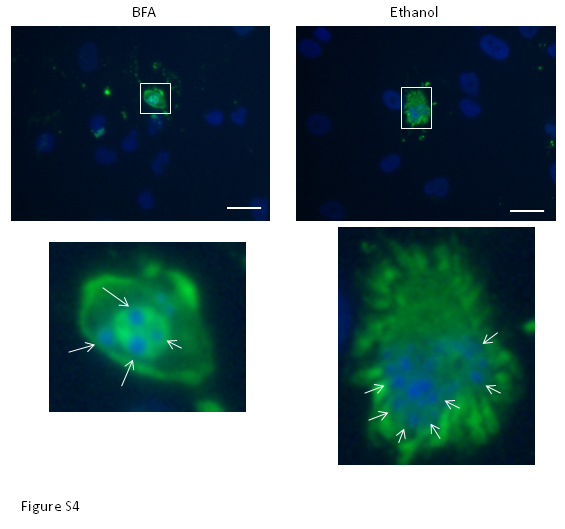

Supplement: Figure S4 — Induction of cleaved caspase 3 by ethanol and Brefeldin A. Ethanol (1%) and Brefeldin A (BFA; 10 µM) were used as positive controls to induce cleaved caspase 3 expression in the astrocytes. The white boxed area has been enlarged to highlight the destruction of the nucleus, which has been reduced to numerous small vesicle like structures. This pattern of nuclear damage and cleaved caspase 3 was identical to that induced by IL1β and TNFα. Scale bar is 50 µm. (TIF) [file pone.0084269.s004.tif]
